# Impacts of uORF codon identity and position on translation regulation

**DOI:** 10.1093/nar/gkz681

**Published:** 2019-08-08

**Authors:** Yizhu Lin, Gemma E May, Hunter Kready, Lauren Nazzaro, Mao Mao, Pieter Spealman, Yehuda Creeger, C Joel McManus

**Affiliations:** 1 Department of Biological Sciences, Carnegie Mellon University, Pittsburgh, PA 15213, USA; 2 Department of Cell and Tissue Biology, University of California, San Francisco, CA 94143, USA; 3 Roche Sequencing Solutions, Santa Clara, CA 95050, USA; 4 Center for Genomics and Systems Biology, New York University, New York, NY 10003, USA; 5 Molecular Biosensor and Imaging Center, Carnegie Mellon University, Pittsburgh, PA 15213, USA; 6 Computational Biology Department, Carnegie Mellon University, Pittsburgh, PA 15213, USA

## Abstract

Translation regulation plays an important role in eukaryotic gene expression. Upstream open reading frames (uORFs) are potent regulatory elements located in 5′ mRNA transcript leaders. Translation of uORFs usually inhibit the translation of downstream main open reading frames, but some enhance expression. While a minority of uORFs encode conserved functional peptides, the coding regions of most uORFs are not conserved. Thus, the importance of uORF coding sequences on their regulatory functions remains largely unknown. We investigated the impact of an uORF coding region on gene regulation by assaying the functions of thousands of variants in the yeast YAP1 uORF. Varying uORF codons resulted in a wide range of functions, including repressing and enhancing expression of the downstream ORF. The presence of rare codons resulted in the most inhibitory YAP1 uORF variants. Inhibitory functions of such uORFs were abrogated by overexpression of complementary tRNA. Finally, regression analysis of our results indicated that both codon identity and position impact uORF function. Our results support a model in which a uORF coding sequence impacts its regulatory functions by altering the speed of uORF translation.

## INTRODUCTION

As an essential step in gene expression, mRNA translation is highly regulated by complex interactions between *cis*-acting sequences and *trans*-acting factors. Translation is regulated primarily at the initiation stage, in which ribosomes select start codons and initiate protein synthesis. Eukaryotic translation initiation occurs through directional scanning. Pre-initiation complexes (PICs), comprised of 40S small ribosomal subunits and numerous initiation factors, assemble at mRNA 5′ ends and scan the mRNA in a 5′ to 3′ direction until a suitable start codon is encountered (1). As a consequence of directional scanning, the presence of mRNA structures and regulatory sequences between the 5′ 7-meG cap and the main ORF coding sequence can affect initiation efficiency. Upstream open reading frames (uORFs) are short coding sequences that function as potent *cis*-acting regulators of translation initiation. While most studied uORFs repress translation at downstream main ORF (mORF) coding sequences, some act as stress-dependent enhancers through re-initiation at downstream mORF start codons (2,3).

Translation of yeast GCN4 mRNA provides a classic example of stress-dependent uORF regulatory control (3). GCN4 is a master regulator of starvation stress responses, whose expression is regulated by four uORFs. Translation of the most 5′ of these, uORF1, allows frequent resumption of directional scanning by post-termination 40S subunits. Under rich growth conditions, when levels of the eIF2 ternary complex are high, re-initiation at one of the remaining 3 uORFs leads to translation termination without production of the Gcn4 protein. Stress conditions reduce the availability of ternary complex, which results in leaky scanning past uORFs 2–4 and re-initiation at the GCN4 mORF start codon. Thus, the GCN4 uORF1 enhances mORF translation under stress by insulating against the inhibitory uORFs 2–4 (3). A similar delayed initiation mechanism controls expression of the mammalian transcription factor ATF4, which also controls mammalian starvation responses (4). Indeed, uORFs are found in the transcript leaders of many transcription factors, including the yeast AP1-like stress response genes YAP1 and YAP2 (5). The YAP1 uORF has a relatively minor impact on production of YAP1p and is not required for stress resistance (5) In contrast, the two uORFs of YAP2 strongly inhibit production of Yap2p. Thus, uORFs have a wide range of regulatory impacts on many genes, including stress-responsive transcription factors.

The regulatory functions of some uORFs are influenced by their coding sequences. A small number of uORFs have been found to encode regulatory proteins. For example, the arginine attenuator peptide (AAP) is encoded by a uORF upstream of the *Neurospora crassa* arg-2 gene (6,7). Under growth conditions rich in arginine, the AAP causes ribosomes translating this uORF to stall and, as a result, decreases translation of arg2 mRNA. Other functional coding sequences have been identified in mammalian uORFs, including a peptide in the AZIN1 gene. This gene encodes the antizyme inhibitor protein, which functions in regulating intracellular polyamine levels. Under high-polyamine concentrations, the AZIN1 uORF peptide causes ribosome stalling (8). Other examples of uORF encoded peptides may function to regulate a wide array of cellular processes (9). However, the extent to which most uORF coding regions affect regulatory functions remains largely unknown.

Several recent studies have found that rare codons can also cause ribosomes to stall in protein coding genes (10–14). Furthermore, some combinations of adjacent codons (dicodons) have even stronger effects on protein production that depend on their specific order (15,16). Previously, a rare codon in a uORF upstream of the *Xenopus laevis* Cx41 gene was found to contribute to strong uORF repression (17). The impact of this rare codon was observed when placed at a particular location close to the uORF start codon, suggesting it may impede scanning by upstream 48S pre-initiation complexes. Similarly, the addition of a rare leu codon in the AAP peptide encoded by the uORF of *N. crassa* arg-2 increased arginine-mediated repression. (18). Genetic analysis also implicated the identity of the last codon in the yeast GCN4 uORF1 in translation re-initiation, as alleles with A/U rich codons more often complimented a *gcn4*-Δ mutant strain (19). Despite the importance of codons in translation and hints of their uORF roles, their functional impact on uORF regulation has not been systematically investigated. Traditional analyses of uORF functions involve comparing luciferase expression from uORF-containing and uORF-lacking reporters, which has limited the scale of uORF studies.

Here, we introduce a massively parallel reporter assay for high-throughput testing of uORF functions (FACS-uORF). We used FACS-uORF to examine the impact of coding sequence variants in a uORF upstream of the YAP1 gene in yeast. The wild-type YAP1 uORF functions as a mild expression enhancer in our assay system. By testing thousands of YAP1 uORF variants, we found that both codon identity and position affect uORF regulatory functions. The presence of non-optimal codons and inhibitory codon pairs led to more repressive uORF functions, while optimal codons were correlated with enhancer activity. The repressive nature of rare codons in uORFs was eliminated by overexpression of complementary tRNA, indicating that slow uORF translation was responsible for inhibitory uORFs. Our results support a model in which uORF translation speed plays an important role in their control of downstream ORF translation.

## MATERIALS AND METHODS

### Plasmid library construction and transformation

YAP1 uORF constructs were assayed using a reporter plasmid expressing both YFP and mCherry (*pGM-YFP-mCherry)* (20). All primers used are listed in (Supplementary Table S1) The YAP1 transcript leader was cloned between the GPM1 promoter, including 19 nt of GPM1 transcript leader and an XmaI site (AAACAAACACACATATTACCCCGGG), and YFP (see Supplementary Figure S1). A library of 4,096 uORF variants were cloned using an oligo containing degenerate bases ‘MRN’ in the third, fourth and fifth codons of the Yap1 uORF (ATGAACMRNMRNMRNTTTTAG; ATG_Template). A second library of 4,096 uORF start codon mutant plasmids was cloned using a corresponding oligo carrying a non-functional AGG start codon (AGGAACMRNMRNMRNTTTTAG; AGG_Template). ATG_Template and AGG_Template were polymerase chain reaction (PCR) amplified for 20 cycles with primers MRN3_PCR1_F and MRN3_PCR1_R. The PCR product was gel extracted and PCR amplified for 15 cycles using primers MRN3_PCR2_F and MRN3_PCR2_R. This second PCR product was gel extracted and PCR amplified for 25 cycles using primers MRN3_PCR3_F and MRN3_PCR3_R. The resulting PCR product was column purified, digested with the XmaI and BglII, gel purified and ligated into the vector *pGM-YFP-mCherry*. Plasmid libraries were transformed into *Escherichia coli*, and ∼40,000 colonies were collected from each set to maximize variant representation. Plasmid libraries were extracted using a QIAGEN Maxiprep kit as per manufacturers’ instructions.

A second library of 368 Yap1 uORF variants was constructed containing 11 previously reported repressive codon pairs (16). Oligos Dicodon_ATG and Dicodon_AGG were PCR amplified for 20 cycles using primers MRN_PCR1_F and MRN_PCR1_R. PCR products were gel extracted and amplified an additional 30 cycles using primers MRN_PCR2_F and MRN_PCR2_R. The PCR products were purified using AMPure XP magnetic beads as per manufacturers’ instructions, digested with XmaI and BglII, and cloned into the vector *pGM-PTH761-YFP-BglII*. The plasmid libraries were transformed into *E. coli* and collected as described above.

A total of 400 μl of BY4741 (MATa his3Δ1 leu2Δ0 met15Δ0 ura3Δ0) (21) competent cells were transformed with 2 μg of a plasmid library pool containing the MRN and dicodon libraries, along with wild-type and AGG-mutant Yap1 constructs using the Frozen-EZ Yeast Transformation II Kit™ (Zymo Research) as per manufacturers’ instructions. To test the transformation efficiency, 10 μl of cells were plated on minimal media -URA plates and incubated for 48 h at 30°C. Colonies were then counted to ensure at least 100,000 individual transformants were obtained. The remaining cells were incubated overnight in 30 ml of -URA media shaking at 30°C. The next day, the cells were added to 200 ml of -URA media and incubated overnight shaking at 30°C.

### FACS

Yeast carrying the reporter library were grown overnight in -URA media, restarted in 50 ml of -URA media at OD_600_ = 0.1–0.2, and grown shaking at 30°C to OD_600_ ∼ 0.8. Immediately before cell sorting, 12 ml of cells were pelleted and flash frozen for later RNA extraction, and 1 ml of cells were pelleted and frozen for DNA extraction. The remaining culture was gated on forward and side-scatter for cell size, and cells were sorted on the YFP/mCherry ratio using a FACSVantage Digital Cell Sorter. The 488 and 532 nm lasers were used to excite YFP and mCherry, respectively. A 530/30 filter was used for YFP in combination with a 532 notch filter to remove 532 nm incidental laser scatter, and a 620/60 filter was used for mCherry. YFP emission can excite mCherry fluorescence. To offset this, BD FacsDiva compensation software was employed to resolve spectral overlap, using yeast expressing only mCherry (*PTH761-CEN-mCherry_v4*) or YFP (*pGM-PTH761-YFP)*. For each of eight sort bins, 100,000 cells were deposited into culture tubes containing 5 ml of URA- glucose media, and grown overnight shaking at 30°C. The next morning, the YFP/mCherry fluorescence ratio for each bin was measured on a Tecan M1000 plate reader to verify sorting and adjust the fluorescence values of each bin for replicate comparisons.

### FACS-uORF assay—sequencing library preparation

RNA libraries were prepared from total RNA extracted from the yeast prior to sorting. Total RNA was extracted using acid-phenol:chloroform, with glass bead grinding, precipitated and resuspended in 250 μl of water. A total of 5 μg of RNA was treated with four units of TURBO™ DNase (Thermo Fisher Scientific) for 30 min at 37°C. The DNase was removed with one round of acid-phenol:chloroform extraction, and RNA was recovered using a RNA Clean & Concentrator™ column (Zymo Research) in 50 μl of water. Reverse transcription was performed using 1 μg of total RNA in a 20 μl reaction (1× Superscript IV buffer, 2 μM reverse primer (Rev1), 0.5 mM dNTPs, 20 mM DTT, 20 units SUPERase-In™ (Thermo Fisher Scientific) and 200 units SuperScript™ IV RT (Thermo Fisher Scientific)) incubated at 50°C for 30 min. A total of 1 μl of reaction mixture containing cDNA was PCR amplified using primers that anneal upstream and downstream of the Yap1 uORF (Fwd1-a, Fwd1-b, Fwd1-c and Rev1), in a 50 μl reaction (1× Q5 polymerase buffer, 0.5 μM primers, 0.2 mM dNTPs, 1 unit of Q5^®^ Hi-Fi DNA polymerase). Each primer includes five to seven random bases to add sequence complexity for Illumina sequencing. The amplification conditions were 98°C for 30 s, followed by 10 cycles of 98°C for 30 s, 59°C for 30 s and 72°C for 30 s, and one cycle of 72°C for 2 min. PCR products were purified with 1.5× AMPure XP beads, and resuspended in 30 μl of water. An additional 10 cycles of PCR were performed using primers that included Illumina sequences and 6 nt barcodes (Fwd2 and RPF-Tag-Rev) containing 2 μl of the first PCR product, 1× Q5 polymerase reaction buffer, 0.5 μM each primer, 0.2 mM each dNTP and 0.4 units of Q5^®^ High-Fidelity DNA polymerase, in a 20 μl reaction. The amplification conditions were 98°C for 30 s, followed by 14 cycles of 98°C for 10 s, 64°C for 10 s and 72°C for 30 s and then one cycle of 72°C for 2 min. The PCR products were cleaned up using a 1.5× concentration of AMPure XP beads, and resuspended in 15 μl of water.

Yap1 uORF transcript leaders were sequenced from each bin (1–8) and from the original unsorted population. The DNA libraries were prepared using the same PCR conditions and primers as described above for the RNA libraries. Briefly, 1 ml of liquid culture from each of the eight sorted bins, and from the original unsorted culture, was pelleted. The media was removed, and the cells were frozen for several hours to aid in cell lysis. The yeast plasmid DNA was extracted using the Zymoprep™ Yeast Plasmid Miniprep II kit (Zymo Research) according to the manufacturers’ instructions and 50 ng of plasmid was used as a template for the first PCR. All of the libraries were pooled and sequenced on an Illumina MiSeq for 150 cycles in each direction.

### Computational analysis of FACS-uORF

Paired-end sequencing reads of FACS-uORF were first merged with FLASh (version 1.2.11) (22), and aligned to our custom designed YAP1 uORF construct reference with Bowtie2 (version 2.2.4) (23). A custom python script count.py (Supplementary File 1) was used to filter the bowtie2 output .sam files and count for perfectly mapped reads for each designed uORF construct. The normalized YFP/mCherry value for each uORF variant $v$${Y_v}$ was calculated as the weighted average of Tecan measured YFP/mCherry value for each fluorescence-associated cell sorting (FACS)-sorted bin: ${Y_v} = \mathop \sum \limits_{i = 1}^n \ \frac{{{F_{v,\ i}}}}{{\mathop \sum \nolimits_{j\ = \ 1}^n {F_{v,\ j}}}}\ {Y_i}$, where $n$ is the total number of bins ($n\ = \ 8$ in our experiment), ${Y_i}$ is the Tecan measured YFP/mCherry level for bin $i$, and ${F_{v,\ i}}$ is the fraction of DNA-seq reads count for variant $v$ in bin $i$, normalized by the fraction of cells in each bin (relative to total sorted cells in the population): ${F_{v,\ i}} = \frac{{{\rm CellCoun}{t_i}}}{{\mathop \sum \nolimits_{j = 1}^n {\rm CellCoun}{t_j}}}\ \centerdot \frac{{{\rm ReadsCoun}{t_{v,\ i}}}}{{{\rm ReadsCoun}{t_{{\rm total},\ i}}}}$ (See Supplementary Figure S2). RNA-seq and DNA-seq read counts in unsorted fraction were used to calculate transcription levels. We verified that replicates are highly correlated, then used the mean value of replicates for further analysis. Normalized read count values are available in Supplementary Table S2.

### tRNA overexpression

tRNA overexpression vectors (2 μ LEU2 plasmid (empty vector), 2 μ LEU2 tR(ACG)D-A34U, 2 μ LEU2 tP(UGG)F-U34C, 2 μ LEU2 tV(CAC)D) were kindly provided by the Grayhack lab (16). The vectors were transformed into *Saccharomyces cerevisiae* BY4741 by selection on -LEU medium. YAP1 uORF variant reporter constructs containing 6 different repressive dicodon pairs (CGA-CGA, CGA-CCG, CCG-CGA, GGA-CCG, GTG-CGA and CGA-GTG) were cloned using PCR from template oligos (e.g. Yap1-Dicodon-CGA-CGA, Supplementary Table S1). Briefly, 1 μM of oligo template was PCR amplified using the primers MRN3_PCR1_F and MRN3_PCR1_R for 20 cycles. The PCR products were gel purified and used as a template for a second PCR using the primers MRN3_PCR2_F and MRN3_PCR2_R for 30 cycles. The PCR products were gel purified, digested with XmaI and BglII, and cloned into the vector *pGM-PTH761-YFP-BglII-mCherry*. YAP1 reporter constructs were transformed into the tRNA overexpression strains and in BY4741 (control) and selected on minimal media lacking leucine and uracil. Individual colonies for each construct were grown in triplicate overnight at 30°C in 5 ml of liquid -LEU/-URA minimal media (or -URA minimal media for the BY4741 only control) and the fluorescence ratio of YFP over mCherry was measured using a Tecan M1000 plate reader.

## RESULTS

### FACS-uORF—an assay for simultaneous analysis of thousands of uORF sequence variants

**Figure 1.**
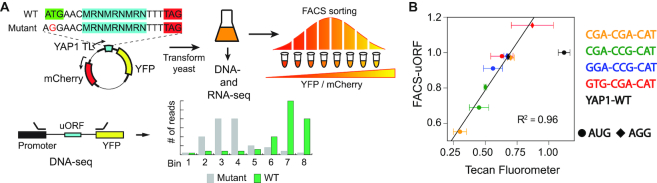
(**A**) The FACS-uORF massively parallel reporter assay. A library of YAP1 uORF reporter plasmids was generated containing 16 variable codons in the middle three positions. uORF regulatory functions were assayed by comparing YFP protein expression from AUG (wt) and AGG (no-start mutant) plasmid constructs, normalized by mCherry (internal control). The plasmids were transformed into yeast. A portion of yeast culture was saved for bulk high-throughput DNA-seq and RNA-seq. The rest were FACS sorted into eight bins based on their YFP/mCherry ratio. Plasmid DNA was recovered from each bin and sequenced on an Illumina MiSeq. YFP/mCherry ratios were estimated from the distribution of plasmids across FACS bins. (**B**) Fluorometer validation. Selected uORFs were subcloned, transformed into yeast and YFP/mCherry ratios of monoclonal cultures were measured in triplicate using a Tecan Fluorometer and compared to FACS-uORF estimates. uORFs containing CGA, CCG and GTG codons were drawn from the additional 368 variants cloned later in the SKR-SSR-VRT ‘dicodon’ library. Excluding the YAP1-WT AUG construct, which was anomalously overrepresented in the reporter library, Tecan and FAS-uORF measurements are highly correlated (R^2^ = 0.96; 0.64 with YAP1-WT), suggesting FACS-uORF provides accurate expression estimates.

We tested the effects of thousands of YAP1 uORF sequence variants on gene expression using FACS-uORF (Figure 1A), a high-throughput uORF functional assay inspired by massively parallel reporter assays of gene expression (15,24,25). We designed 4,096 different uORF variants in the YAP1 transcript leader by replacing the third, fourth and fifth codons of the YAP1 uORF with ‘MRN’, where M is A/C, R is A/G and N is any nucleotide. In addition, we also included corresponding transcript leaders where the uORF start codon was mutated to the non-functional AGG (26). YFP levels were determined from the distribution of each transcript leader construct in bins of cells sorted by the YFP/mCherry ratio via FACS. The gene-regulatory impact of each uORF variant was assayed by comparing YFP levels from wild-type and AGG-mutant uORF transcript leaders. YFP measurements were highly reproducible, as seen from a comparison of two biological replicates (Supplementary Figure S3A; *R*2 = 0.96), and correlated well with Tecan M1000 measurements of YFP/mCherry ratios from 10 subcloned transcript leaders (Figure 1B).

### Coding sequence variation creates a wide range of uORF effects on main ORF expression

**Figure 2.**
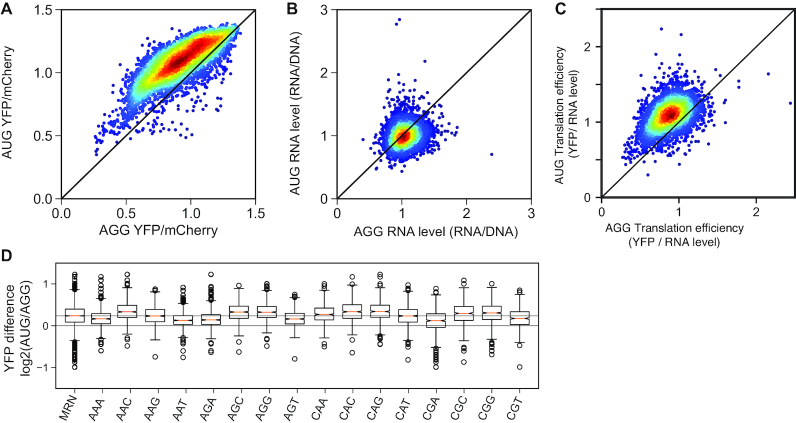
Synthetic YAP1 uORF variants alter reporter expression in a codon-dependent manner. (**A**) Scatter plot of estimated YFP levels from wild-type (AUG) and non-functional (AGG) uORF mutants. Most YAP1 uORFs upregulate the reporter protein. (**B**) Scatter plot of transcript abundance from uORF and mutant uORF reporters, corrected for relative plasmid representation (RPM RNA/RPM DNA). uORF containing reporters have similar transcript levels (∼4.7% lower on average) compared to AGG mutants. (**C**) Scatter plot of translation efficiency (YFP/RNA) for all AUG and AGG reporters. (**D**) Boxplots show the distribution of uORF effects on YFP/mCherry levels from all 4,096 uORF constructs (MRN), and uORF constructs containing each of the 16 codons encoded by MRN in at least one of the three varied positions. Codon variants impart a wide range of uORF functions, ranging from 2-fold repression to 2-fold enhanced YFP production. Data are presented as the log2 ratio of YFP/mCherry from the uORF-containing (AUG) and corresponding uORF mutant (AGG) reporter plasmids. uORF variants containing CGA codons were among the most repressive.

Having established FACS-uORF, we investigated the impact of codon variants on uORF function. The wild-type YAP1 uORF functions as an enhancer in our reporter system, since the presence of this uORF results in a 1.65-fold increase in YFP protein levels (measured by Tecan fluorometry) compared to an AGG start codon mutant lacking the uORF (Figure 1B, (20)). Similarly, the majority of codon variant uORFs are also enhancers, as YFP expression is higher from most AUG-uORF containing transcript leaders than from corresponding AGG-mutant leaders (Figure 2A). While enhancer uORFs have been described previously (20,27,28), they are considered unusual. Consequently, we also assayed mRNA levels from AUG-uORF and AGG-mutant constructs using YFP-targeted RNA-seq. Transcription levels were estimated as the ratio of construct RNA levels to relative plasmid representation in transformed yeast (RNA/DNA ratio). Despite having consistently higher protein levels produced by AUG-uORF constructs, there was little difference in relative RNA levels (RNA/DNA) of AUG-uORF constructs compared to AGG-mutant constructs (Figure 2B and Supplementary Figure S4). In fact, AUG-uORF constructs had slightly lower RNA levels (4.7% lower on average) than AGG-mutant constructs. Consequently, the majority of AUG-uORF containing plasmids resulted in higher YFP mORF translation efficiency than AGG-mutant plasmids (for possible mechanisms, see the ‘Discussion’ section). We conclude that variant YAP1 uORFs affect YFP reporter expression mainly by altering translation efficiency.

We next evaluated the effects of ‘MRN’ codons on uORF functions (Figure 2D). The 4,096 uORF coding-sequence variants had wide-ranging effects on YFP expression, from 2-fold enhancers to 2-fold repressors. uORF variants containing codons AAA, AAT, AGA, AGT, CGA and CGT were all associated with relatively lower enhancer activity, including some variants that converted the YAP1 uORF into repressors. CGA containing variants had the strongest inhibitory effect compared to other codons (*P*-value = 2.5e-33). The Arg CGA codon is extremely non-optimal in yeast (29), and it is the only codon decoded by an inefficient inosine–adenosine wobble pair (30). Previous work has implicated CGA codons in No-Go mRNA Decay (NGD) (15,16,31), resulting from collisions of translating 80S ribosomes. Although YAP1 uORF variants can only accommodate a single 80S ribosome, CGA containing variants showed a 10.7% decrease in mRNA levels on average, two times lower than the average over all variant uORFs (*P* = 3.97e-13; *t*-test). These results indicate that variations in uORF coding sequence alter their impact on gene expression.

### Codon pairs have order-dependent influences on uORF regulatory functions

**Figure 3.**
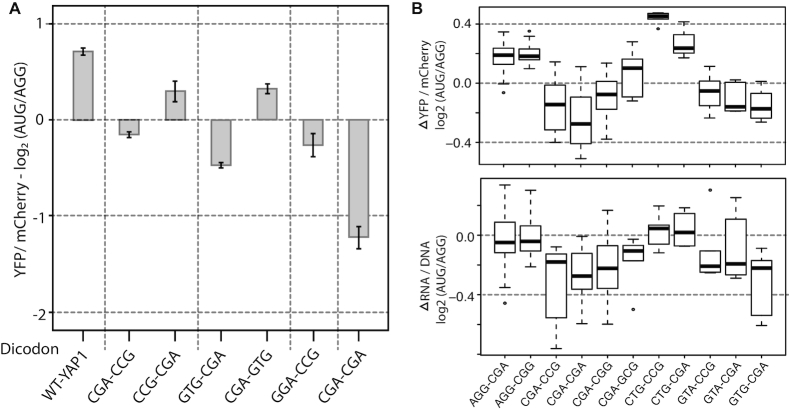
Effects of inhibitory codon pairs on uORF regulation. (**A**) Four inhibitory dicodons (Gamble *et al.*, (16) were cloned into codons 2 and 3 of the AUG- and AGG-uORF reporter constructs, and YFP/mCherry ratios were measured in triplicate using a plate fluorometer. Barplot shows the ratio of YFP expression in AUG- versus AGG-uORF plasmids. For each of the dicodons, inclusion of the forward (A/B) pair resulted in inhibitory uORFs. Reversed order (B/A) pairs were also cloned for two dicodons (CCG-CGA and CGA-GTG). In both cases, the resulting uORFs were no longer inhibitory. (**B**) FACS-uORF was used to assay 368 additional uORF coding variants, including 11 additional inhibitory dicodons from Gamble *et al.* Boxplots show the distribution of uORF regulatory effects at the protein (top) and RNA (bottom) level for each of these inhibitory dicodons. These distributions depict the regulatory impact of all uORF variants containing the dicodon, in both codons 2 and 3 and codons 3 and 4. uORFs containing 6 of these 11 reduced YFP expression at both the protein and RNA levels.

Recent studies identified adjacent codon pairs that act in concert in translation regulation, inhibiting protein synthesis more strongly than single rare codons (16). The regulatory impact of some dicodon pairs depended on codon order. We asked whether codon order influenced the regulatory functions of uORFs. Our MRN library covers 120 unique pairs of codons, located at two positions (dicodon 1–2 and dicodon 2–3). Among dicodon 1–2 pairs, 50 have significant differences in uORF repression when altering the codon order (*P*-adj < 0.05;Supplementary Figure S5A). In contrast, only two dicodon two–three pairs have significant differences in uORF activity upon codon order switching (Supplementary Figure S5B). Three codon pairs in our uORF library (CGA-CGG, AGG-CGG and AGG-CGA) were previously reported to have order-dependent effects on mORFs. Of these, CGA-CGG and AGG-CGG display a similar order dependence in uORFs (*P*-value = 4e-6 and *P*-value = 1.6e-4, respectively), while AGG-CGA showed no significant order dependence. We cloned two additional previously reported order-dependent codon pairs (GUG-CGA and CGA-CCG) into the YAP1 uORF. Both pairs showed the expected order dependence, as uORFs bearing the forward (A/B) dicodons were repressors, while those bearing reverse order (B/A) dicodons were enhancers (Figure 3A).

To more fully investigate the impact of inhibitory codon pairs, we cloned a second uORF library containing 368 additional coding variants in the YAP1 uORF (SKR-SSR-VRT; S = C/G, K = G/T, R = A/G and V = A,C,G), including eleven of the seventeen previously reported inhibitory dicodons reported in (16). Six of these previously reported inhibitory dicodons were associated with repressor activity when located in the YAP1 uORF (Figure 3B). Interestingly, uORFs encoding all six of these repressive dicodon pairs resulted in a 10–15% decrease in median RNA levels, while those harboring the non-repressive dicodons did not (Figure 3B). We conclude that, consistent with previous studies on mORF expression, some dicodon pairs have order-dependent effects on uORF functions. Furthermore, the order dependence was more common for codon1-2 pairs than for codon2-3 pairs (Supplementary Figure S5). At last, we find that the most inhibitory dicodons affect expression by reducing mRNA levels.

### uORF codon effects depend on the balance between tRNA supply and demand

**Figure 4.**
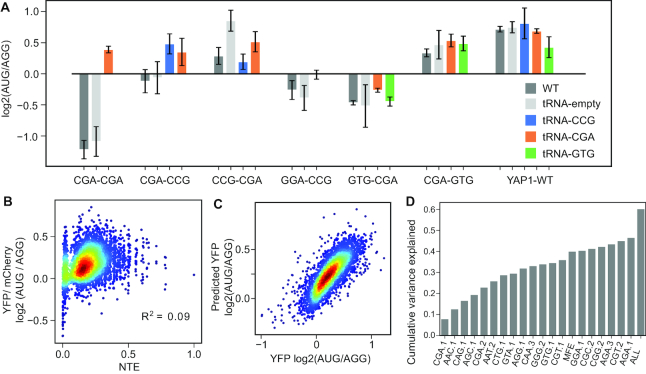
Influence of tRNA abundance, codon identity and position on uORF regulatory effects. (**A**) Selected uORFs’ translational regulatory effects were assayed by comparing YFP expression from reporter constructs containing wild-type (AUG) and mutant (AGG) uORFs using a Tecan fluorimeter. The uORF regulatory effect is expressed as the log2 ratio. Overexpression of tRNA complimentary to the rare CGA and CCG codons reduced inhibition by uORFs harboring those codons, while overexpression of tRNA complimentary to the more common GTG codon had no effect. (**B**) The uORF’s normalized TE score (NTE) is positively correlated with its regulatory effect. (**C**) Lasso regression model of variant uORF function. The scatterplot shows a comparison of predicted vs measured uORF effects. The model explains 58.6% of the variance in uORF regulatory effects. (**D**) Variance importance plot. The top 20 important predictors were shown from left to right. The first position CGA is most predictive among all predictors.

Previous studies indicated that codon effects on expression from main ORFs are correlated with the abundance of complementary tRNA (32,33), and that the effects of repressive dicodon pairs could be alleviated by overexpressing tRNA complementary to the corresponding codons (16). We hypothesized that overexpression of complementary tRNA would similarly alleviate translational repression by uORFs bearing repressive codons. Consistent with this, overexpression of a CGA tRNA relieved the repressive effect of YAP1 uORFs harboring a CGA codon (Figure 4A). Strikingly, variants encoding the CGA-CGA dicodon were converted from 2-fold repressors to ∼1.3-fold enhancers. The rescue effect was also observed in uORF constructs encoding a single CGA codon, but to a lesser extent. Similarly, overexpression of a CCG tRNA relieved repression by uORFs bearing CGA-CCG and GGA-CCG dicodons. These results indicate that the regulatory effects of YAP1 variant uORFs are dependent on the relative abundance of tRNA complementary to uORF codons.

We next asked whether the functions of other YAP1 uORF codon-variants are associated with the abundance of corresponding tRNA. We compared the average nTE score, a measurement of tRNA expression level normalized by the corresponding codon's expression level (estimated by mRNA abundance and codon usage) (10), to the protein level change caused by uORF translation. As shown in Figure 4B, the uORF nTE score is positively correlated with protein level changes, indicating tRNA abundance is involved in uORF codon's regulatory function. uORFs containing codons read by relatively rare tRNA are more repressive, while those decoded by relatively common tRNA have higher enhancer functions. However, nTE scores explain only 9% of the variance in uORF regulatory functions, suggesting the total codon optimality of uORFs is not enough to explain the regulatory effects caused by uORF codons.

### Regression modeling identifies codon identity and position as key predictors of uORF function

The results above show that both codon identity and position affect the regulatory functions of uORF variants on translation of downstream YFP. However, a quantitative measurement of codons’ contribution is desired for us to understand the importance of codon's regulatory function. We used linear multiple regression modeling with lasso feature selection to estimate the extent to which protein level changes caused by uORF translation can be explained by codon usage and codon position. Codon usage in uORFs was encoded as a binary variable where I(mrn, i) = 1 indicates position i in the triple MRN library is codon mrn. This encoding gave us 63 variables for feature selection and regression modeling. With 10-fold cross-validation, the lasso regression analysis selected 56 variables out of the 63 variables and explains 59.0% of the variance of the log_2_ fold change of YFP between AUG uORF and AGG uORF (Supplementary Figure S6A). The performance of the regression model increased slightly to 59.5% variance explained if we also include predicted minimum free energy of the 5′UTR RNA secondary structure as a predictor (Figure 4C). In contrast, using the codon identity without considering codon position as predictor only explains 40.6% of the variance (Supplementary Figure S6B), using amino acid composition and position only explains 19.7% of the variance (Supplementary Figure S6C). The most important predictors are shown in Figure 4D. Consistent with our previous results, the presence of CGA codons in the first position was the most influential predictor. Indeed, many of the highly ranked predictors are codon choices on the first position while codons in the second and third position of the varied region had less influence on uORF function.

## DISCUSSION

**Figure 5.**
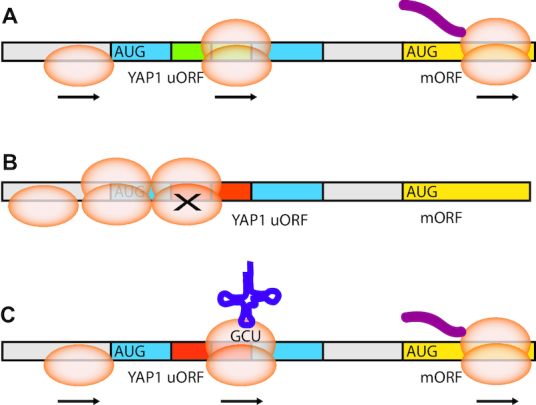
Model of codon impact on uORF regulatory functions. (**A**) uORFs bearing optimal codons are translated efficiently, leading to rapid clearance of 80S ribosomes from mRNA transcript leaders. (**B**) The presence of non-optimal, ‘rare’ codons and dicodon pairs within uORF coding regions leads to 80S ribosome stalling. Stalling impedes scanning by upstream 43S PICs, reducing the flow of ribosomes to the downstream main ORF. (**C**) Changes in levels of charged tRNA alter the rate of uORF translation, allowing increased scanning and initiation efficiency at the downstream mORF. As tRNA levels are dependent on environmental conditions (yeast) and cellular differentiation (metazoan), uORF codons may impart similar dependence on translation regulation.

Although codon usage is well known to regulate translation of main ORFs, there have been no systematic studies of the functional importance of uORF coding regions. Thus, very little is known regarding the impact of codon usage in uORF functions. Here, we introduce the FACS-uORF assay and systematically compare the regulatory impact of thousands of codon variants of the YAP1 uORF. We find that both the position and identity of codons within this uORF impact its regulatory function, leading to a wide range of enhancer and repressor activities. uORFs with common codons were more likely to be enhancers, while uORFs with rare codons were associated with repressive functions. These results support a model in which the speed of uORF translation impacts their regulatory functions (Figure 5). Our study has many important implications for translational control, as discussed in the following.

Previous studies identified conserved peptide-encoding uORFs (cpuORFs) that stall ribosomes in response to the presence of small-molecule metabolites (8,34,35). The resulting inhibition of downstream main ORF translation likely occurs by preventing loading and/or scanning of additional 43S translation PICs. Rare codons and inhibitory codon pairs are also known to cause ribosome stalling in main ORFs, leading to ribosome collisions that induce No-Go Decay (16,31). We found uORF variants bearing rare codons and inhibitory codon pairs repressed translation of downstream main ORFs. As with cpuORFs, it seems likely that these codons and dicodons increase uORF repression by stalling ribosomes and decreasing the rate of PIC loading and scanning. Based on previous studies, the fact that several inhibitory dicodons resulted in reduced mRNA levels suggests that stalling on uORF rare codons and dicodons also induce No-Go Decay. Consistent with this, we showed that overexpression of complementary tRNA reversed the inhibitory effects of uORFs carrying inhibitory dicodons, resulting in functional enhancers. In combination with the correlation between uORF repressiveness and normalized Translation Efficiency estimates (NTE), these results suggest that, as with main ORFs, uORFs are sensitive to changes in levels of charged tRNA.

In addition to codon identity and levels of complementary decoding tRNA, our results indicate that the exact location of rare codons within uORFs influences their impact on gene regulation. Our regression model revealed that variants in the first position had a more substantial influence on uORF functions than variants in other positions. Similarly, dicodons 1–2 were more sensitive to order than dicodons 2–3. Several mechanisms may contribute to this position dependence. In our assay, this first position is the third codon in the uORF. Ribosomes at such an early stage in translation may be more sensitive to the availability of charged tRNA, while those that have elongated further may be more robust. Alternatively, stalling at this first position may more strongly impact loading and scanning of upstream 43S PICs. Ribosomes stalled at the first variable codon would thus be perfectly placed to increase initiation at a near-cognate CUG codon 29 nt upstream by creating a roadblock to scanning. Similar mechanisms have been recently reported for increased near-cognate initiation due to roadblocks created by RNA structure and cpuORF stalling (8,36). Thus, the position dependence of YAP1 uORF codon variants could reflect general sensitivity of ribosomes at early stages of elongation or the specific sequence environment upstream of the uORF.

Our results also suggest a mechanism that could impart condition and/or tissue-specificity to uORF functions. Yeast tRNA levels vary in response to stress (37). Similarly, tRNA levels vary among mammalian tissues (38,39), and are differentially transcribed in proliferating and differentiated cells. Such changes in tRNA levels are correlated with the codon demand imparted by the corresponding mRNA expressed in those cell types and would most likely alter the speed of codon translation in a tissue-dependent manner. If rare codons increase the repressive nature of natural uORFs in metazoans (e.g. *X. laevis* Cx41 (17)), variation in tRNA compositions among conditions and tissues may result in uORFs with condition- and tissue-specific regulatory effects. Similarly, changes in tRNA levels could alter uORF functions when cells enter a state of uncontrolled proliferation during oncogenesis. In this case, codon-engineered uORFs could be used to increase tissue-specificity of experimental mRNA therapeutics (40,41). Furthermore, natural variation in human uORF coding regions could have tissue-specific effects on health. Importantly, uORFs are found much more frequently in human transcript leaders than in yeast (∼50% of genes versus ∼13%) and are more re-initiation permissive (42). The large number of human uORFs underscores the potential influence of codon usage on human gene regulation. Thus, future studies are needed to examine how varying tRNA pools might alter the functions of natural and designer uORFs.

One unusual aspect of our study is that the wild-type YAP1 uORF increases expression of the YFP reporter. While most uORFs repress downstream ORFs, a few enhancer uORFs have been previously identified (20,27,43). Such enhancer uORFs seem at odds with the scanning model of translation initiation. However, several potential mechanisms could result in enhancer uORF activity. uORF translation could remodel transcript leader structure or remove bound proteins, clearing the way for other PICs to more efficiently proceed to the mORF start codon. Alternatively, uORFs might increase protein production by controlling the flow of traffic on the downstream mORF. Occasional uORF translation could reduce the density of ribosomes on the mORF, resulting in fewer collisions and reduced No-Go Decay. These mechanisms would allow uORF translation to optimize the density of ribosomes on the downstream main ORF.

Another possibility is that enhancer uORFs function in a manner similar to GCN4 uORF1, by insulating scanning PICs from more repressive uORFs downstream through delayed re-initiation(3). The YAP1 uORF shares sequence and structural features known to promote re-initiation by GCN4 uORF1 (44,45), and is known to allow frequent re-initiation (5). In contrast to GCN4, the YAP1 transcript leader lacks additional downstream AUG-uORFs. However, three interceding UUG triplets, though individually poor recruiters of scanning PICs, could together comprise the repressive uORFs that would be insulated by the YAP1 uORF. In this case, the relatively short spacing (65 nt) between the YAP1 uORF and the mORF start codon might allow reniitiation-competent scanning PICS to skip the interceding UUG codons and initiate at the downstream AUG mORF start codon, even during exponential growth in non-stress conditions (as in the current study). Functioning as an enhancer would further require mechanisms that protect the YAP1 mRNA from uORF-induced nonsense-mediated decay. Notably, the YAP1 uORF has a weak Kozak sequence (UUGUGC**AUG**A), and its transcript leader is protected from Nonsense Mediated Decay (NMD) through interactions with the RNA binding protein Pub1p (46). While the mechanisms underlying enhancer uORFs remain unclear, computational modeling of translation initiation may provide useful insights (47).

uORFs have been found in more than half of mammalian genes and have crucial regulatory roles in many biological pathways. This prevalence of uORFs underscores the need for quantitative models that predict uORF function. Our lasso regression model, using only codon identity and position, explained 58% of the variance among uORF codon variants. Our results showed that the order of adjacent codon pairs can impact uORF functions, suggesting this may account for some of the unexplained variation among uORFs in our assay system. Additionally, recent work found that the energy needed to unwind mRNA structure surrounding a uORF start codon can influence the rate of uORF initiation (48). Thus, some of the unexplained variance may reflect variations in transcript leader structure resulting from uORF coding sequence differences. Regardless of the sources underlying the unexplained variance, our results clearly implicate codon usage in modulating uORF activity. Future applications of FACS-uORF and similar approaches are needed to build complete predictive models of natural and designer uORF functions.

## DATA AVAILABILITY

Raw Illumina FASTQ sequencing data of plasmids from each FACS-sorted bin have been submitted to NCBI under project accession number PRJNA556437. Plasmid count data are included in Supplementary File 1.

## Supplementary Material

gkz681_Supplemental_Files
