# Supplementary material for: Impacts of uORF codon identity and position on translation regulation
Source: Nucleic Acids Res. 2019 Aug 8;47(17):9358–67. doi: 10.1093/nar/gkz681 (PMC6755093; doi:10.1093/nar/gkz681)
Supplement: gkz681_Supplemental_Files [file gkz681_supplemental_files.zip › Supplemental-Material-revised.pdf]

Supplementary Material

YML007W-chrXIII;253762-253783

AAACACACATATTACCCCGGGATTAAGCACAGTACCTTTACGTTATATATAGGATTGGTGTTTAGCTTTTTTTCCTG
AGCCCCTGGTTGACTTGTGCATGAACACGAGCCATTTTTAGTTTGTTTAAGGGAAGTTTTTTGCCACCCAAAACGTT
TAAAGAAGGAAAAGTTGTTTCTTAAACCATGAGATCTAAAGGTGAAGAATTATTCACTGGTGTTGTCCCAATTTTG
GTTGAATTAGATGGTGATGTTAATGGTCACAAATTTTCTGTCTCCGGTGAAGGTGAAGGTGATGCTACTTACGGTA
AATTGACCTTAAAATTAATTTGTACTACTGGTAAATTGCCAGTTCCATGGCCAACCTTAGTCACTACTTTAGGTTATG
GTTTAATGTGTTTTGCTAGATACCCAGATCATATGAAACAACATGACTTTTTCAAGTCTGCCATGCCAGAAGGTTAT
GTTCAAGAAAGAACTATTTTTTTCAAAGATGACGGTAACTACAAGACCAGAGCTGAAGTCAAGTTTGAAGGTGAT
ACCTTAGTTAATAGAATCGAATTAAAAGGTATTGATTTTAAAGAAGATGGTAACATTTTAGGTCACAAATTGGAAT
ACAACTATAACTCTCACAATGTTTACATCACTGCTGACAAACAAAAGAATGGTATCAAAGCTAACTTCAAAATTAGA
CACAACATTGAAGATGGTGGTGTTCAATTAGCTGACCATTATCAACAAAATACTCCAATTGGTGATGGTCCAGTCT
TGTTACCAGACAACCATTACTTATCCTATCAATCTGCCTTATCCAAAGATCCAAACGAAAAGAGAGACCACATGGTC
TTGTTAGAATTTGTTACTGCTGCTGGTATTACCCATGGTATGGATGAATTGTACAAATAG .....

Figure S1. Sequence of the wildtype YAP1 reporter construct. As described in the methods, the YAP1 transcript leader was cloned between the GPM1 promoter and YFP. The 5' end is the transcription start site provided by the GPM1 promoter, followed by 21 nucleotides of GPM1 transcript leader (black font). The YAP1 transcript leader is shown in red font, and the uORF is highlighted in blue, with the variant region underlined. The YFP main ORF is shown in black font and highlighted in yellow. The YAP1 mORF and YFP ORF have the same first five nucleotides (ATGAG).

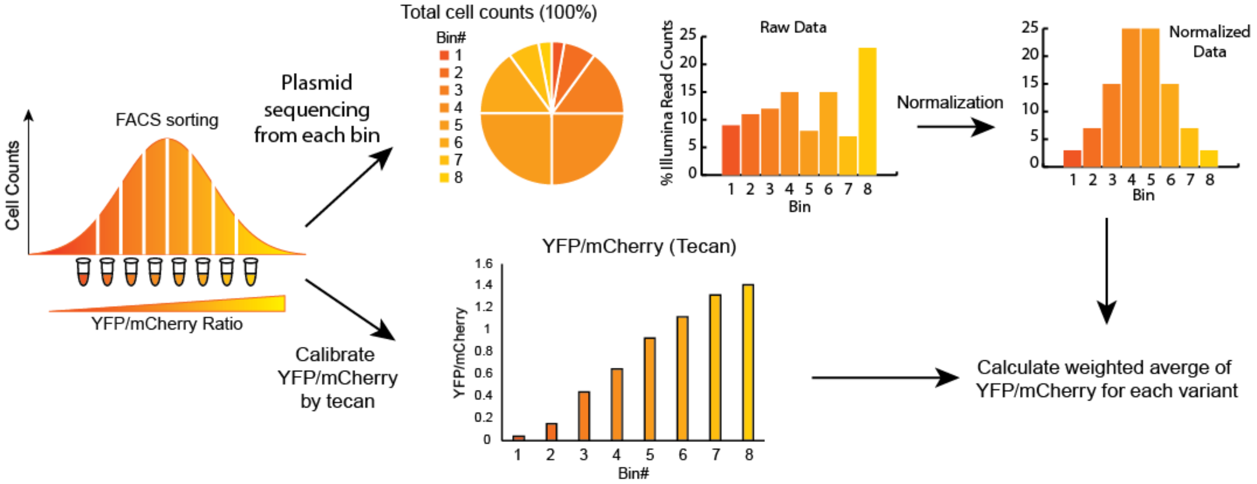

Figure S2. FACS-uORF data analysis pipeline. Illumina sequencing data were aligned to the respective constructs to generate a table of raw hits to each construct from each bin. Ideally, the number of Illumina read-hits per bin should be proportionate to the number of cells per bin from FACS-sorting. For example, if 10% of the cells were sorted into bin 1, 10% of the total illumina read-hits should align to that bin. Raw hits were normalized by fractional downsampling, to ensure this proportionality. After this normalization step, the weighted average YFP/mCherry expression value was calculated for each uORF construct using the per-bin YFP/mCherry values that were assayed from each sorted bin of yeast after overnight growth in YEPD. For full details, see the Materials and Methods section.

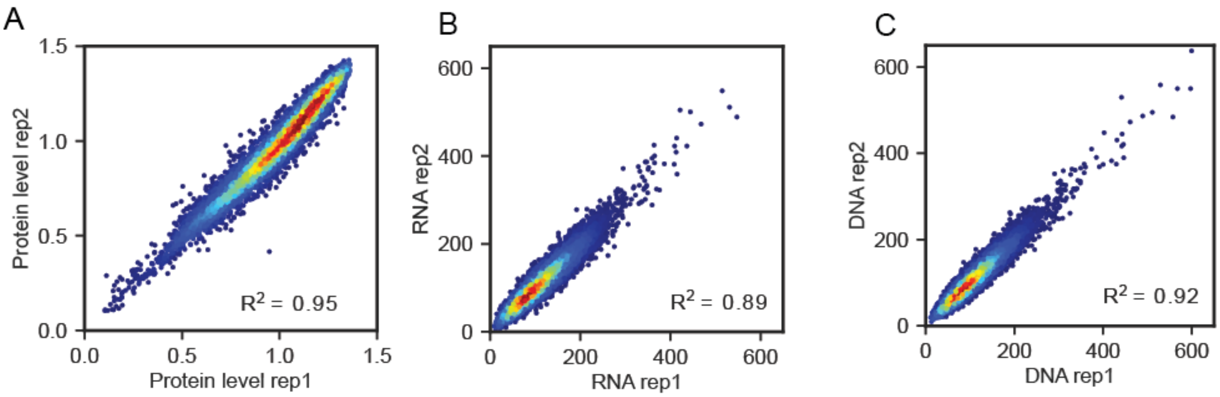

Figure S3. Scatterplots show correlations between replicates 1 and 2. (A) Replicates of protein level measured by FACS-uORF. (B) Replicates of RNA levels measured by RNA-seq. (C) Replicates of transformed plasmids DNA level measured by DNA-seq.

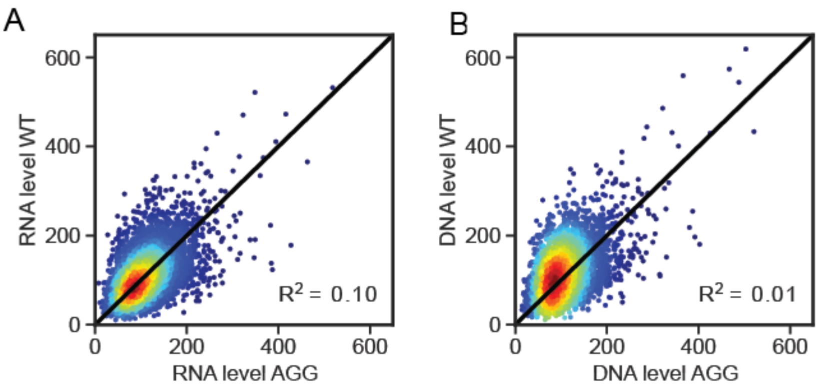

Figure S4. Scatterplots comparing AUG-uORFs to AGG-uORFs. (A) RNA levels. (B) DNA levels. Both scatterplots show low r-squared value, and uORF is not likely an enhancer regulating RNA levels.

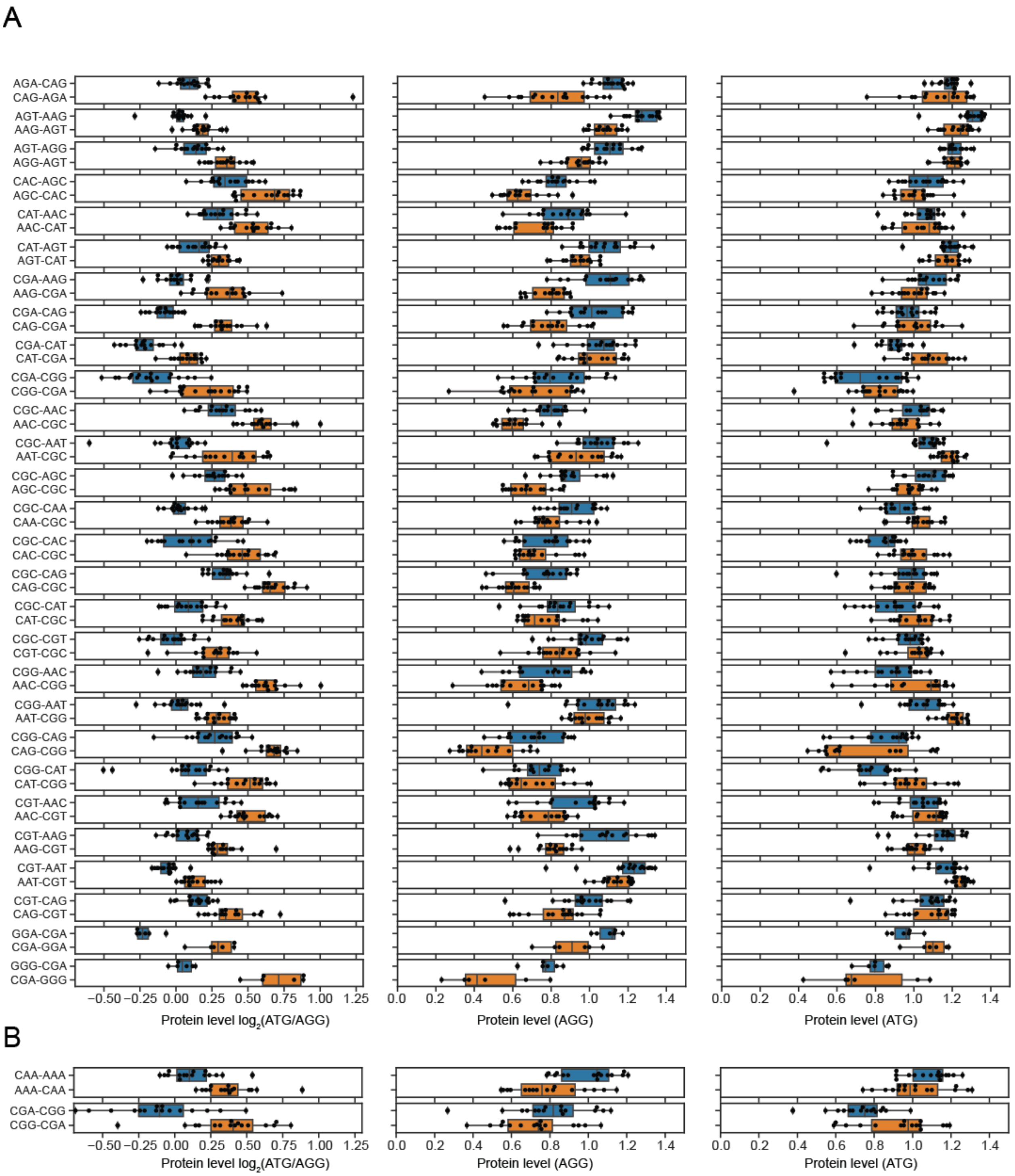

Figure S5. Boxplots summary of codon pairs that shows different regulatory effect when reversed. (A) 28 position 1 and 2 codon pairs out of 128 total pairs shows adjusted p-val < 0.01 (B) Only 2 out of 120 position 2 and 3 codon pairs shows adjusted p-val < 0.05. (CAA-AAA p-adj = 0.03, CGA-CGG p-adj = 0.01).

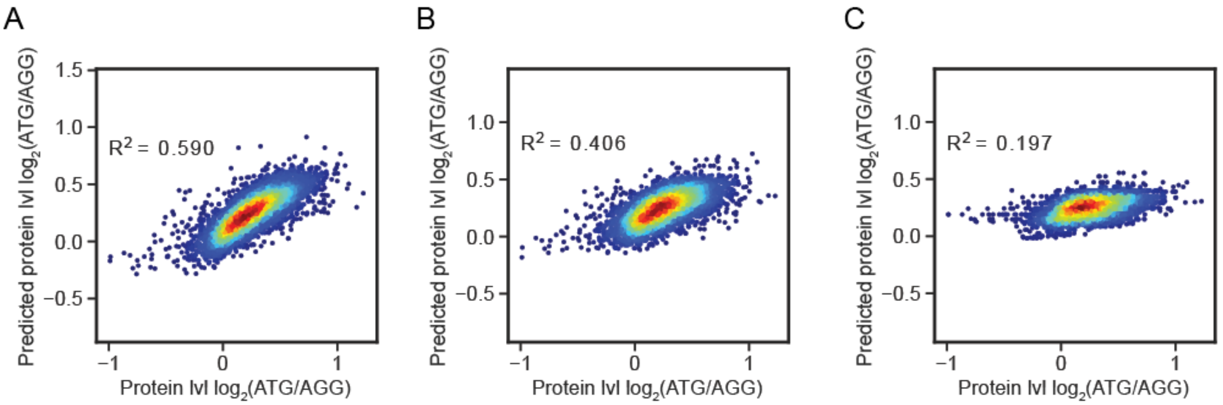

Figure S6. Scatterplots of predicted protein level and measured protein level when using different regression model. (A) Using only codon and codon positions in regression model. (B) Using only codon counts and 5'UTR MFE in regression model. (C) Using only amino acid and amino acid positions in regression model.

Table S1. Primers used in this study

Name	Sequence 5'-3'
ATG_Template	5’_CCTGAGCCCCTGGTTGACTTGTGCATGAACMRNMRNMRNTTTTAGTTTGTTTAAGGGAAGTTTTTGCCACCCAAAACGTTTAAAGAAGG_3'
AGG_Template	5’_CCTGAGCCCCTGGTTGACTTGTGCAGGAACMRNMRNMRNTTTTAGTTTGTTTAAGGGAAGTTTTTGCCACCCAAAACGTTTAAAGAAGG_3’
MRN3_PCR1_F	5’_CAGTACCTTTACGTTATATATAGGATTGGTGTTTAGCTTTTTTTCCTGAGCCCCTGGTTG_3’
MRN3_PCR1_R	5’_TTCTTCACCTTTAGATCTCATGGTTTAAGAAACAACTTTTCCTTCTTTAAACGTTTTGGG_3’
MRN3_PCR2_F	5’_CTTAATAATCCAAACAAACACACATATTACCCCGGGACAGTACCTTTACGTTATATATAG_3’
MRN3_PCR2_R	5’_TCTAATTCAACCAAAATTGGGACAACACCAGTGAATAATTCTTCACCTTTAGATCTCATG_3’
MRN3_PCR3_F	5’_ATTACCTTCCTTTGTAATTTTTTTTGTAATTATTCTTCTTAATAATCCAAACAAACACAC_3’
MRN3_PCR3_R	5’_CACCGGAGACAGAAAATTTGTGACCATTAACATCACCATCTAATTCAACCAAAATTGGG_3’
Dicodon_ATG	5’_CCTGAGCCCCTGGTTGACTTGTGCATGAACSKRSSRVRTTTTTAGTTTGTTTAAGGGAAGTTTTTGCCACCCAAAACGTTTAAAGAAGG_3’
Dicodon_AGG	5’_CCTGAGCCCCTGGTTGACTTGTGCAGGAACSKRSSRVRTTTTTAGTTTGTTTAAGGGAAGTTTTTGCCACCCAAAACGTTTAAAGAAGG_3’
Fwd1-a	5’_CACTCTTTCCCTACACGACGCTCTTCCGATCTNNNNNNCTGAGCCCCTGGTTGACTT_3’
Fwd1-b	5’_CACTCTTTCCCTACACGACGCTCTTCCGATCTNNNNNNNCTGAGCCCCTGGTTGACTT_3’
Fwd1-c	5’_CACTCTTTCCCTACACGACGCTCTTCCGATCTNNNNNCTGAGCCCCTGGTTGACTT_3’
Rev1	5’_TTCAGACGTGTGCTCTTCCGATCTNNNNNNCCTTTAGATCTCATGGTTTAAG_3’
Fwd2	5’_AATGATACGGCGACCACCGAGATCTACACTCTTTCCCTACACGACGC_3’
RPF-Tag-Rev	5’_CAAGCAGAAGACGGCATACGAGATXXXXXXGTGACTGGAGTTCAGACGTGTGCTCTTCCG_3’
Yap1-Dicodon-CGA-CGA	5'_CCTGAGCCCCTGGTTGACTTGTGCAKGAACCGACGACATTTTTAGTTTGTTTAAGGGAAGTTTTTGCCACCCAAAACGTTTAAAGAAGG_3'
Yap1-Dicodon-CGA-CCG	5'_CCTGAGCCCCTGGTTGACTTGTGCAKGAACCGACCGCATTTTTAGTTTGTTTAAGGGAAGTTTTTGCCACCCAAAACGTTTAAAGAAGG_3'
Yap1-Dicodon-CCG-CGA	5'_CCTGAGCCCCTGGTTGACTTGTGCAKGAACCCGCGACATTTTTAGTTTGTTTAAGGGAAGTTTTTGCCACCCAAAACGTTTAAAGAAGG_3’
Yap1-Dicodon-GGA-CCG	5'_CCTGAGCCCCTGGTTGACTTGTGCAKGAACGGACCGCATTTTTAGTTTGTTTAAGGGAAGTTTTTGCCACCCAAAACGTTTAAAGAAGG_3'
Yap1-Dicodon-GTG-CGA	5'_CCTGAGCCCCTGGTTGACTTGTGCAKGAACGTGCGACATTTTTAGTTTGTTTAAGGGAAGTTTTTGCCACCCAAAACGTTTAAAGAAGG_3'
Yap1-Dicodon-CGA-GTG	5'_CCTGAGCCCCTGGTTGACTTGTGCAKGAACCGAGTGCATTTTTAGTTTGTTTAAGGGAAGTTTTTGCCACCCAAAACGTTTAAAGAAGG_3'

Table S2. Expression data from 4,465 YAP1 uORF variant reporter plasmids. Columns include the normalized read count for each expression bin (Bin1-8), normalized RNA expression read counts (reads per million), relative plasmid read counts from the transformed yeast library (reads per million), an RNA level estimate (RNA / DNA), estimated YFP / mCherrry levels based on the distribution of each plasmid in the eight sorted bins, and an estimated translation efficiency (YFP / RNA level).

Supplementary File 1. This zip archive contains the python and R scripts used to analyze the FACS-uORF data from raw Illumina FASTQ files (count.py) and generate the expression data shown in Table S1. The archive includes a README.txt file describing the included scripts.
